# Impact of diabetes mellitus on mortality in patients with acute exacerbation of chronic obstructive Pulmonary disease: a meta-analysis and systematic review

**DOI:** 10.3389/fendo.2026.1838301

**Published:** 2026-05-13

**Authors:** Dongmei Zhang, Hongbo Zhou, Xiujuan Yang, Dajian Xia

**Affiliations:** 1 Department of Emergency, The People’s Hospital of Dazu, Chongqing, Chongqing, China; 2 Department of Respiratory and Critical Care Medicine, The People’s Hospital of Dazu, Chongqing, Chongqing, China; 3 Department of Nursing, The People’s Hospital of Dazu, Chongqing, Chongqing, China

**Keywords:** acute exacerbation of chronic obstructive pulmonary disease, diabetes mellitus, meta-analysis, mortality, systematic review

## Abstract

**Background:**

As one of the most prevalent comorbidities in chronic obstructive pulmonary disease (COPD), diabetes mellitus has drawn growing attention for its potential impact on clinical outcomes in acute exacerbation of COPD (AECOPD). However, the association between diabetes and mortality risk in AECOPD remains controversial. This meta-analysis synthesized available evidence to quantify the effect of diabetes on mortality among AECOPD patients, aiming to provide an evidence-based foundation for clinical risk stratification and individualized intervention.

**Methods:**

We systematically searched PubMed, Embase, Cochrane Library, Web of Science, CNKI, and Wanfang databases up to March 2026 for cohort or case-control studies investigating the association between diabetes and mortality in AECOPD patients. Two researchers independently conducted literature screening, data extraction and quality evaluation. The Newcastle-Ottawa Scale (NOS) was used to assess risk of bias. Meta-analysis was performed using Stata 14.0 software. The hazard rations (HR) was used to combine the effect size, and corresponding 95% confidence interval (CI) was computed.

**Results:**

A total of 9 studies were included, involving 23,883 participants. In the univariate model, diabetes increased the mortality risk of patients with AECOPD (HR = 1.45, 95%CI (1.19,1.77), P < 0.0001), whereas no significant association was observed in multivariate analysis (HR = 1.50, 95% CI (0.45, 5.00), P = 0.513). Subgroup analysis showed that diabetes increased mortality risk in hospitalized AECOPD patients but not in those admitted to intensive care units. A significant association was found in case-control studies but not in cohort studies, and in studies with a sample size > 1000 but not in smaller studies.

**Conclusions:**

This meta-analysis demonstrates inconsistent associations between diabetes and mortality in AECOPD patients, which may be confounded by methodological factors and study design. Current evidence does not support diabetes as an independent risk factor for mortality in AECOPD. Further well-designed, adequately powered prospective cohort studies with rigorous adjustment for confounders are warranted to clarify the true prognostic impact of diabetes on AECOPD patients.

## Introduction

Chronic obstructive pulmonary disease (COPD) is a prevalent chronic respiratory disorder characterized by persistent airflow limitation (1). It has the characteristics of high incidence rate, high disability rate and high mortality rate (1, 2). According to the Global Burden of Disease Study, COPD ranks as the third leading cause of death worldwide, imposing a substantial burden on healthcare systems (2). Acute exacerbation of COPD (AECOPD) is defined as an acute worsening of respiratory symptoms beyond normal daily variation that necessitates a change in treatment plan. It is a major driver of COPD progression, irreversible lung function decline, impaired quality of life, and death (3, 4). According to statistics, the in-hospital mortality of AECOPD patients ranges from 2% to 11%, and long-term mortality remains markedly elevated after acute exacerbation (5, 6). Therefore, identifying the predictive factors for the mortality risk of AECOPD patients is of great significance for clinical risk stratification, early intervention, and improving outcomes.

With population aging and shifting patterns of metabolic diseases, diabetes has emerged as one of the most common comorbidities in COPD patients. The impact of concurrent diabetes on AECOPD outcomes has attracted increasing research interest. Epidemiological studies have shown that the prevalence of diabetes among COPD patients is approximately 15%-17%, significantly higher than in the general population (7). There is a complex bidirectional relationship between diabetes and COPD: A complex bidirectional interaction exists between diabetes and COPD: on the one hand, diabetes may accelerate COPD progression by inducing systemic inflammation, impairing immune function, increasing susceptibility to infection, and promoting airway remodeling (8, 9). On the other hand, chronic hypoxia, systemic inflammation, and repeated glucocorticoid use in COPD elevate the risk of metabolic disorders and new-onset diabetes (8, 9).

However, there is still no consensus among existing studies regarding whether diabetes independently increases the mortality risk of AECOPD patients. Some retrospective cohort studies report significantly higher in-hospital and long-term mortality in diabetic AECOPD patients compared with non-diabetic patients (10, 11). Conversely, other studies fail to detect an independent association, suggesting that the prognostic effect of diabetes may be confounded by age, disease severity, comorbidity burden, and other factors (12, 13). Furthermore, substantial heterogeneity exists across studies in patient selection (e.g., hospitalized vs. intensive care unit patients), study design (prospective vs. retrospective cohort, case-control), sample size, and adjustment for confounders, leading to inconsistent and conflicting results. This further leads to differences and controversies in the results. Accordingly, this study systematically reviewed domestic and international literature and performed a meta-analysis to evaluate the impact of diabetes on mortality in AECOPD patients, providing evidence-based support for risk stratification and personalized management of diabetic AECOPD patients in clinical practice.

## Methods

This study was performed in accordance with the Preferred Reporting Items for Systematic Reviews and Meta-Analysis (PRISMA) 2020 guidelines (14, 15).

### Data sources and search strategy

We conducted a search in PubMed, Embase, Cochrane Library, Web of Science, CNKI, and Wanfang Database to collect cohort studies or case-control studies on the association between diabetes and the mortality risk of patients with AECOPD. The search period covered from the establishment of the databases to March 2026. The search was conducted using a combination of Medical Subject Headings (MeSH) terms and free text terms including: “Chronic Obstructive Pulmonary Disease”, “COPD”, “Acute Exacerbation”, “AECOPD”, “Diabetes Mellitus”, “Diabetes”, “Mortality”, “Death”, “Prognosis”,etc. Reference lists of included studies were manually screened to identify additional eligible literature.

### Inclusion and exclusion criteria

Studies were included if they met all the following criteria:

1. Study Design: Cohort study (prospective or retrospective) or case-control study;
2. Participants: adults aged ≥18 years with a clinical diagnosis of AECOPD.
3. Exposure factor: AECOPD patients with diabetes as the exposed group and those without diabetes as the unexposed group;
4. Outcome: Report data related to the mortality risk;
5. The hazard ratio (HR) and its 95% confidence interval (CI) can be obtained, or sufficient raw data can be provided for calculation.

Studies were excluded if they met any of the following criteria:

1. Duplicate publications;
2. Conference abstracts, case reports, reviews, comments or animal experiments;
3. Studies lacking extractable valid outcome data;
4. Studies with an insufficient sample size (< 50 cases);
5. Studies that did not clearly distinguish between the stable stage and the acute exacerbation stage of COPD.

### Data extraction

Two reviewers independently screened literature and extracted data. Discrepancies were resolved by discussion; if consensus could not be reached, a third reviewer arbitrated. All disagreements and resolutions were documented for verification.

Duplicate publications were removed using EndNote. Titles and abstracts were screened initially to exclude irrelevant articles, and full texts were reviewed for final eligibility.

Two researchers independently extracted the following data: (1) Basic information: first author, publication year, country, study design, study period; (2) Patient characteristics: sample size, age, gender; (3) Outcomes: adjusted or unadjusted HR and its 95% CI; (4) Confounder adjustment: preferentially extracted the most comprehensively adjusted effect estimates if both unadjusted and adjusted data were reported; (5) Evaluation results of bias risk and specific details.

### Risk of bias assessment

The Newcastle-Ottawa Scale (NOS) was used to assess the quality of the included cohort and case-control studies. The NOS comprises three domains: selection of study groups, comparability between groups, and outcome or exposure assessment, with a maximum score of 9. Studies with a score of ≥7 were rated as high quality, 5–6 as moderate quality, and ≤4 as low quality. Quality assessment was performed independently by two reviewers, with disagreements resolved by consensus.

### Statistical analysis

Meta-analysis was performed using Stata 14.0 software. Pooled effect sizes were expressed as HR with 95% CI. In view of the fundamental difference in the control of confounding factors between adjusted and unadjusted HR, direct pooling could introduce confounding bias. Therefore, meta-analysis were performed separately for studies reporting multivariable-adjusted HRs and those providing only univariable HRs in the present study. The statistical heterogeneity among the included studies was evaluated using χ2 test combined with I² statistic. If P ≥ 0.1 and I² ≤ 50%, a fixed-effect model was used for the pooling (16). Otherwise, if there was significant heterogeneity, a random-effect model was used for the pooling, and the results were interpreted cautiously. Subgroup analysis was conducted by study design (Cohort study vs. Case-control study), clinical setting (Hospitalization vs. Intensive Care Unit), sample size (> 1000 vs. < 1000) to explore potential sources of heterogeneity. Sensitivity analysis used the elimination method to assess the impact of individual studies on the pooled effect size, to test the robustness of the results. Publication bias was evaluated using funnel plots, Begg’s test, and Egger’s test.

## Results

### Literature searching results

**Figure 1:**
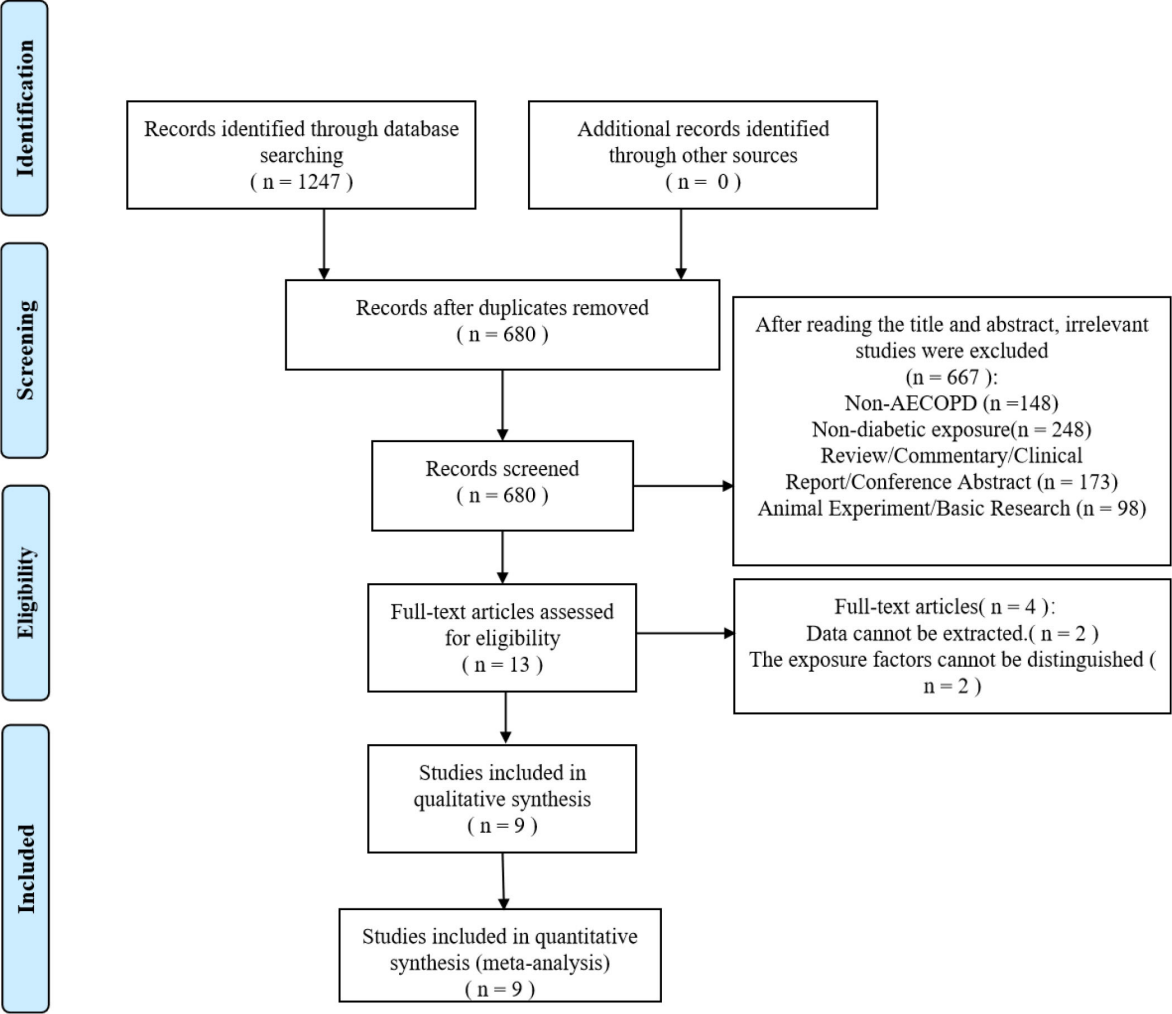
PRISMA flow diagram of study selection process.

The initial search yielded a total of 1247 relevant documents. After removing duplicates, screening titles/abstracts, and reviewing full texts, 9 studies (10–13, 17–21) were finally included in the meta-analysis (**Figure 1**).

### Characteristics of included studies

The basic characteristics of the included studies were detailed in **Table 1**. A total of nine studies published between 2015 and 2026 were included, involving 23,883 participants: 3 were retrospective cohort studies, 3 were prospective cohort studies, and 3 were case-control studies. Six studies enrolled hospitalized patients, and 3 enrolled intensive care unit patients. Four studies had a sample size > 1000, and 5 had a sample size < 1000. All 9 studies had a NOS score ≥8, indicating high methodological quality (**Table 2**).

**Table 1:** Characteristics of included studies.

Study	Country	Study design	Study stage	Clinical setting	Population	Patients(n)	Sex(Male/Female)	Age(Mean),years	Analysis
Chen et al., 2026 (10)	China	Retrospective cohort study	From June 2022 to December 2024	Hospitalization	AECOPD	4292	2126/735	82.5	M
Li et al., 2025(a) (17)	China	Prospective cohort study	From January 1, 2020 to December 31, 2021	Hospitalization	AECOPD	351	229/86	73.97	U
Li et al., 2025(b) (18)	China	Prospective cohort study	From January 1, 2013 to June 30, 2018	Hospitalization	AECOPD	358	236/122	76	U
Xie et al., 2024 (12)	USA	Retrospective cohort study	From June 2008 to December 2019	Intensive Care Unit	AECOPD	606	340/266	72	U
Yayan 2025 (13)	USA	Retrospective cohort study	From June 2008 to December 2019	Intensive Care Unit	AECOPD	5874	3122/2752	68.3	M
Zhu et al., 2026 (21)	China	Prospective cohort study	From January 2023 to December 2023	Intensive Care Unit	AECOPD	878	597/281	Not reported	U
Jiang 2015 (11)	China	Case-control study	From 2005 to 2014	Hospitalization	AECOPD	4624	3298/1326	71.59	U
Liu 2019 (19)	China	Case-control study	From October 2017 to October 2018	Hospitalization	AECOPD	232	159/73	72.73	U
Peng et al., 2021 (20)	China	Case-control study	From September 2017 to January 2021	Hospitalization	AECOPD	6668	5180/1488	76.3	U

AECOPD, Acute exacerbations of chronic obstructive pulmonary disease; U, Univariate; M, Multivariate.

**Table 2:** Risk of bias assessment.

Author (year)	Selection	Comparability	Outcome/Exposure	Total score	Quality
Chen et al., 2026 (10)	★★★★	★★	★★★	9	High
Li et al., 2025(a) (17)	★★★★	★	★★★	8	High
Li et al., 2025(b) (18)	★★★★	★	★★★	8	High
Xie et al., 2024 (12)	★★★★	★★	★★★	9	High
Yayan 2025 (13)	★★★★	★	★★★	8	High
Zhu et al., 2026 (21)	★★★★	★★	★★★	9	High
Jiang 2015 (11)	**★★★★**	★★	★★	8	High
Liu 2019 (19)	★★★★	★★	★★	8	High
Peng et al., 2021 (20)	★★★★	★★	★★	8	High

The number of stars represents the score.

### Meta-analysis

**Figure 2:**
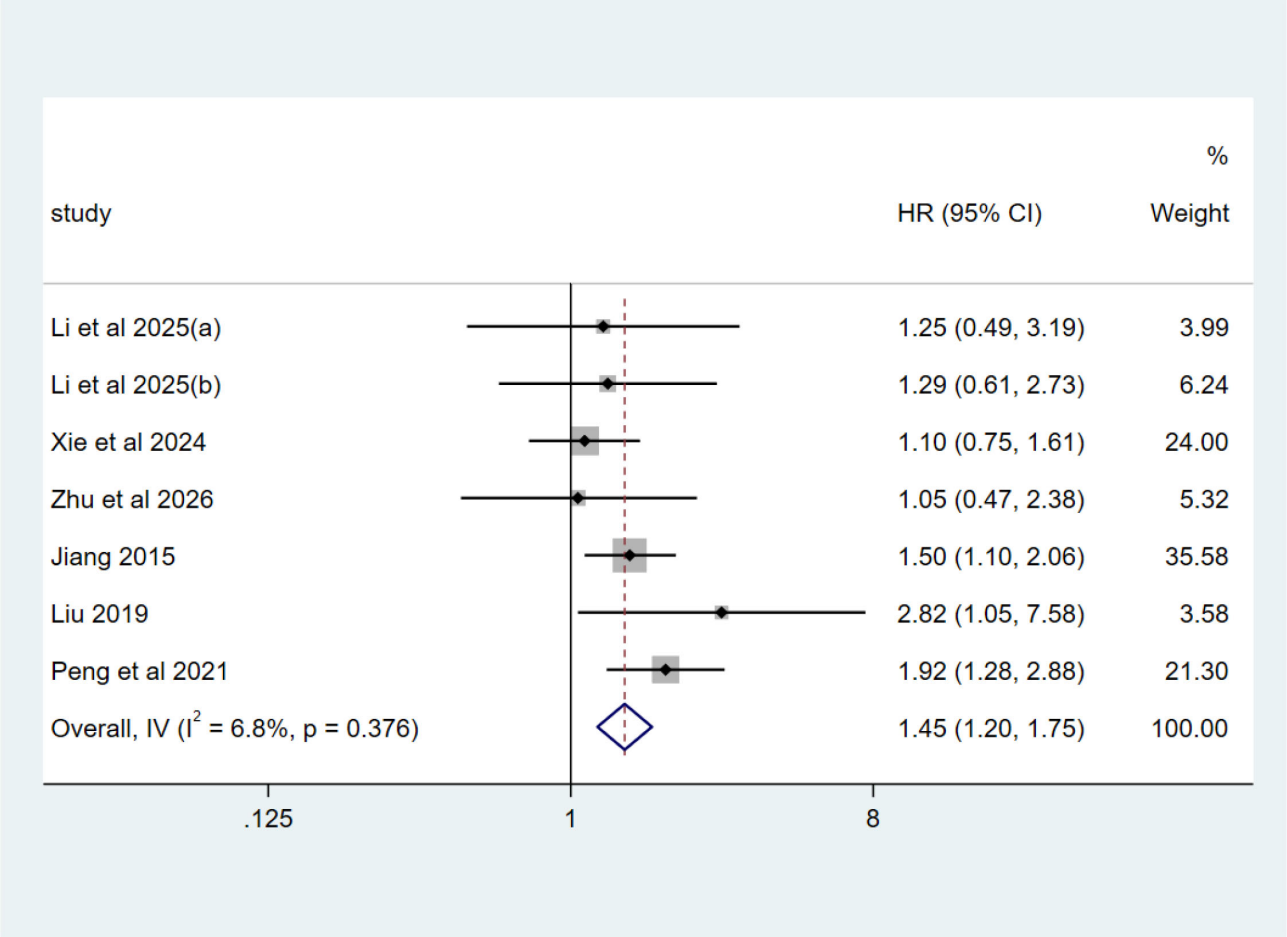
Forest plot of the association between diabetes mellitus and mortality risk in patients with AECOPD summarized by univariate model.

In the univariate model, six studies were included. Heterogeneity test: I^2^ = 6.80%, P = 0.376, a fixed effects model was applied. Diabetes was associated with significantly increased mortality in AECOPD patients (HR = 1.45, 95%CI (1.19,1.77), P < 0.0001) (**Figure 2**).

**Figure 3:**
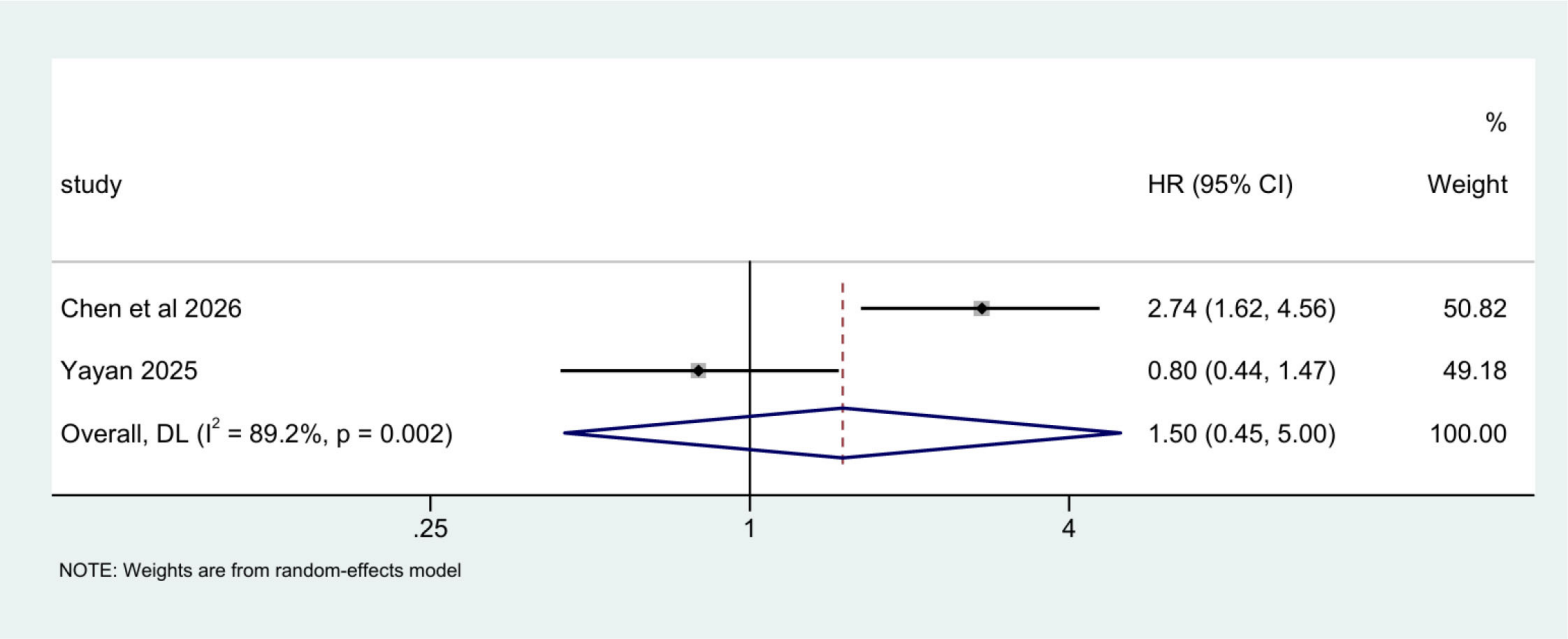
Forest plot of the association between diabetes mellitus and mortality risk in patients with AECOPD summarized by multivariate model.

In the multivariate model, two studies were included. Significant heterogeneity was observed (I² = 89.2%, P = 0.002); a random effects model was used. No significant difference in mortality risk was observed between diabetic and non-diabetic AECOPD patients (HR = 1.50, 95%CI (0.45,5.00), P = 0.513) (**Figure 3**).

### Subgroup analysis

To explore the potential sources of heterogeneity, this study conducted subgroup analysis based on clinical setting, study design and sample size. The results were summarized in **Table 3**.

**Table 3:** Subgroup analysis summarized by univariate model.

Subgroup analysis	Number of studies	Heterogeneity		HR (95%CI)	P Value
		I^2^	P		
Clinical setting					
Hospitalization	4	0%	0.599	**1.63(1.30,2.04)**	**< 0.0001**
Intensive Care Unit	2	0%	0.919	1.09(0.77,1.54)	0.622
Study design					
Case-control study	3	0%	0.374	**1.70(1.34,2.16)**	**< 0.0001**
Cohort study	3	0%	0.975	1.14(0.84,1.53)	0.403
Sample size					
> 1000	4	0%	0.345	**1.65(1.28,2.11)**	**< 0.0001**
< 1000	5	0%	0.525	1.22(0.92,1.63)	0.163

The bold values means the differences were statistically significant.

Subgroup analysis based on clinical setting. Diabetes increased mortality in hospitalized AECOPD patients (HR = 1.63, 95%CI (1.30,2.04), P < 0.0001) but not in intensive care unit patients (HR = 1.09, 95%CI (0.77,1.54), P = 0.622).

Subgroup analysis was conducted based on the type of study design. A significant association was found in case-control studies (HR = 1.70, 95%CI (1.34,2.16), P < 0.0001) but not in cohort and prospective cohort studies (HR = 1.14, 95%CI (0.84,1.53), P = 0.403).

Subgroup analysis was conducted based on the sample size. Diabetes was associated with higher mortality in studies with sample size > 1000 (HR = 1.65, 95%CI (1.28,2.11), P < 0.0001) but not in those with sample size < 1000 (HR = 1.22, 95%CI (0.92,1.63), P = 0.163).

### Sensitivity analysis

**Figure 4:**
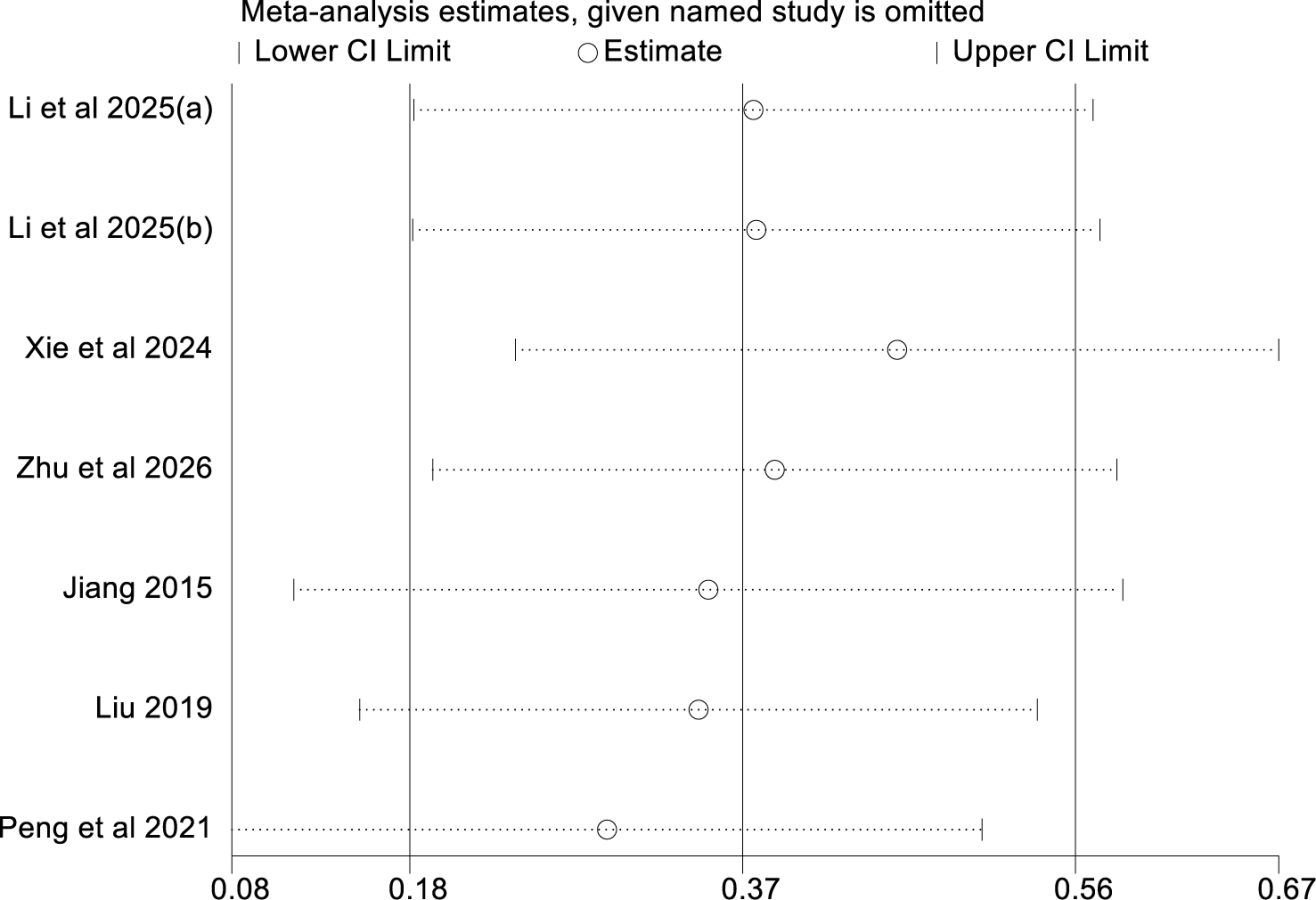
Sensitivity analysis results summarized by univariate model.

Sensitivity analysis was performed using the elimination method. Repeated meta-analysis after omitting each study individually showed that pooled results were robust and not driven by any single study (**Figure 4**).

### Publication bias

**Figure 5:**
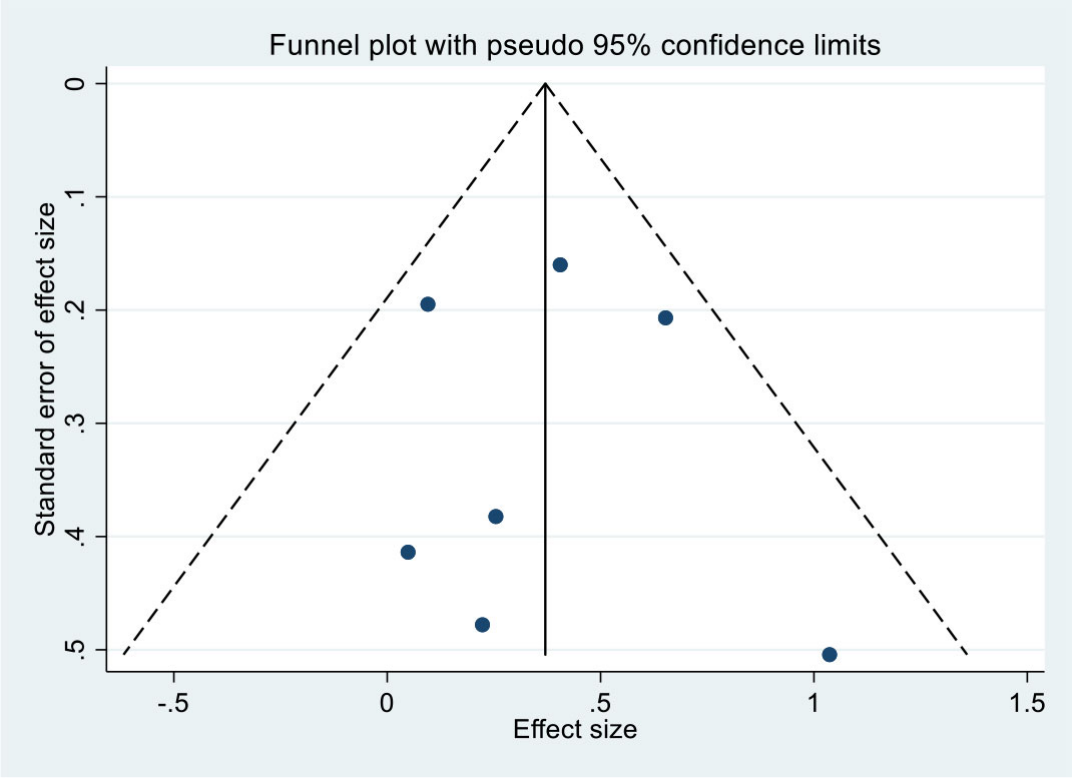
Funnel plot for publication bias assessment summarized by univariate model.

Funnel plots showed an approximately symmetric distribution (**Figure 5**). Begg’s test (P = 1.000) and Egger’s test (P = 0.815) indicated no significant publication bias.

## Discussion

This meta-analysis included 9 studies involving a total of 23,883 patients with AECOPD to systematically evaluate the association between diabetes and the risk of mortality in AECOPD patients. The results demonstrated that diabetes was significantly correlated with an increased mortality risk in the univariable model; however, this association lost statistical significance in the multivariable adjusted model. Subgroup analysis further revealed that diabetes significantly elevated the mortality risk among hospitalized patients, whereas no significant association was observed in intensive care unit patients. An increased mortality risk related to diabetes was detected in case–control studies but not in cohort studies, and a higher mortality risk was identified in studies with a sample size greater than 1,000. Collectively, these findings indicate that the association between diabetes and mortality in AECOPD patients is inconsistent and may be modulated by confounding factors and study design.

The meta-analysis based on univariable models indicated that diabetes may increase the mortality risk in patients with AECOPD. This finding was consistent with the results of many previous studies (10, 11). Diabetes may worsen disease severity and increase mortality risk through multiple pathophysiological mechanisms. Hyperglycemia combined with inflammation impairs lung tissue repair and recovery (22). Furthermore, hyperglycemia also damages pulmonary microcirculation, reducing oxygen delivery and exacerbating hypoxemia-related complications related to hypoxemia in patients with COPD (23). The state of hyperglycemia can induce the body to produce excessive systemic inflammatory responses (8). Studies have shown that the levels of inflammatory markers such as C-reactive protein, interleukin-6, and tumor necrosis factor-αin patients with diabetes are significantly elevated (24). Since AECOPD itself is characterized by intense airway and systemic inflammation, diabetes may exert a synergistic pro-inflammatory effect, triggering an “inflammatory storm” (8, 25). Furthermore, diabetes impairs host immunity by reducing neutrophil chemotaxis and phagocytosis, macrophage activity, and T-cell function, increasing the risk of secondary infection—a major trigger and lethal complication of AECOPD. Infection is the most common trigger of AECOPD and an important cause of death (26, 27). Diabetic micro- and macrovascular complications also impair reserve function of vital organs (heart, kidney), predisposing patients to multiple organ failure during AECOPD (28–30).

Subgroup analysis showed that diabetes significantly increased mortality in hospitalized patients but not in intensive care unit patients (ICU). Intensive care unit patients generally have life-threatening conditions such as respiratory failure, shock, or multiple organ dysfunction; their mortality is mainly determined by acute illness severity and response to organ support, while the prognostic impact of chronic comorbidities like diabetes may be masked (13, 31). Moreover, intensive glucose monitoring and insulin therapy in intensive care units may partially mitigate hyperglycemia-related harm. In contrast, general ward patients have milder acute illness, making the prognostic effect of diabetes more evident.

This study found that there were significant differences in the effect estimates of the association between diabetes and mortality in patients with AECOPD across different research designs. Case-control studies showed a significant association, whereas cohort studies did not. Which means case-control studies are prone to selection and recall bias, which may overestimate the association. Prospective cohort studies, with prospective ascertainment of exposure and outcome, clear temporal sequence, and systematic confounder adjustment, provide more reliable causal inference. Future research should prioritize prospective cohort designs with rigorous confounder adjustment to accurately assess the independent prognostic effect of diabetes.

Studies with sample size > 1000 showed that diabetes significantly increased the mortality risk, while smaller studies did not. This finding is in line with the general pattern of meta-analysis: large-sample studies have higher statistical power to detect modest effect sizes, whereas small-sample studies are prone to false-negative results due to sampling variability. Small studies also have limitations in population representativeness, confounder control, and analytical stability, requiring cautious interpretation.

Notably, diabetes was associated with a significantly elevated mortality risk when HR were extracted from univariable analysis, whereas no significant association was detected in multivariable models. The 95% CI in the multivariate subgroup was extremely wide (0.45 - 5.00), indicating a limited number of included studies and highly unstable results. More importantly, this discrepancy highlights the critical role of confounding factor adjustment in effect size estimation. Patients with diabetes tend to be older and present with a heavier comorbidity burden, including hypertension, coronary heart disease, heart failure, and chronic kidney disease; these comorbidities are well-established independent predictors of mortality among AECOPD patients. Univariable analysis that fail to account for such confounders may overestimate the independent impact of diabetes on mortality. In contrast, multivariable adjustment for relevant confounders enables a more accurate evaluation of the independent prognostic value of diabetes. For instance, Chen et al. (10). did not adjust for mechanical ventilation status and blood glucose levels, which may have overestimated the effect of T2DM. However, in the study by Yayan et al (13), after adjusting for these factors, the significant prognostic effect of T2DM was no longer evident. Accordingly, the crude association between diabetes and mortality observed in the unadjusted meta-analysis is likely driven predominantly by residual confounding bias, rather than the direct independent effect of diabetes itself. The lack of statistical significance in the multivariable subgroup of the present study may be due to the small number of included studies, heterogeneity in the types of adjusted confounders across investigations, and insufficient overall statistical power.

These findings possess valuable clinical implications. First, diabetes can serve as a crucial indicator for risk stratification in patients with AECOPD. For hospitalized AECOPD patients with comorbid diabetes, intensified clinical monitoring and close surveillance for early deterioration signs are recommended. Secondly, glycemic management is essential for optimizing prognostic outcomes in AECOPD patients. Previous studies have demonstrated that in-hospital hyperglycemia, irrespective of a pre-existing diabetes diagnosis, is correlated with adverse clinical outcomes among hospitalized AECOPD patients (31, 32). Accordingly, individualized glycemic control strategies should be optimized for AECOPD patients with diabetes to maintain blood glucose within a reasonable range while preventing hypoglycemic episodes.

Notably, high-quality evidence establishing optimal glycemic targets for AECOPD patients remains scarce, warranting further dedicated investigations. Additionally, ICU clinicians should conduct comprehensive multidisciplinary prognostic assessments based on patients’ overall clinical status, rather than relying solely on a history of diabetes for prognostic judgment and clinical decision-making.

This study has several limitations. First, the overall number of included studies was relatively small, with fewer studies available for certain subgroup analysis. This contributed to wide confidence intervals and low result stability, necessitating cautious interpretation of subgroup findings. Second, several included studies adopted a retrospective design, which is inherently susceptible to selection, information, and confounding bias. Although adjusted effect sizes were preferentially extracted in the present meta-analysis, inconsistencies in the types of confounders adjusted across individual studies may compromise cross-study comparability. Third, raw individual patient data were unavailable, which precluded more granular patient-level analysis. Fourth, quantitative assessment of publication bias was not feasible for subgroups with <10 studies; thus, potential publication bias could not be fully ruled out. Finally, only English and Chinese publications were enrolled in this meta-analysis, which may introduce language bias.

## Conclusion

In conclusion, the present meta-analysis revealed an inconsistent association between diabetes and mortality in patients with AECOPD, which is likely driven by confounding factors. The significant associations observed in univariable analysis and case–control studies were not validated in cohort studies, multivariable adjusted analysis, and ICU patients. Current evidence does not support diabetes as an independent risk factor for mortality among AECOPD patients. Further well-designed prospective cohort studies with adequate sample sizes and rigorous adjustment for confounders are warranted to clarify whether diabetes independently affects the prognosis of AECOPD patients and elucidate the underlying biological mechanisms. Pending definitive evidence becomes available, clinicians and researchers should interpret the prognostic role of diabetes in AECOPD cautiously.

## Data Availability

The datasets presented in this study can be found in online repositories. The names of the repository/repositories and accession number(s) can be found in the article/supplementary material.
